# Synthesis and Applications of Hybrid Polymer Networks Based on Renewable Natural Macromolecules

**DOI:** 10.3390/molecules28166030

**Published:** 2023-08-12

**Authors:** Dariya Getya, Ivan Gitsov

**Affiliations:** 1 Department of Chemistry, State University of New York—ESF, Syracuse, NY 13210, USA; dgetya@syr.edu; 2 The Michael M. Szwarc Polymer Research Institute, Syracuse, NY 13210, USA; 3 The BioInspired Institute, Syracuse University, Syracuse, NY 13244, USA

**Keywords:** sustainable, renewable, natural macromolecules, hybrid networks, cellulose, chitin, alginic acid, gellan gum, lignin

## Abstract

Macromolecules obtained from renewable natural sources are gaining increasing attention as components for a vast variety of sustainable polymer-based materials. Natural raw materials can facilitate continuous-flow production due to their year-round availability and short replenishment period. They also open new opportunities for chemists and biologists to design and create “bioreplacement” and “bioadvantaged” polymers, where complex structures produced by nature are being modified, upgraded, and utilized to create novel materials. Bio-based macromonomers are expected not only to compete with but to replace some petroleum-based analogs, as well. The development of novel sustainable materials is an ongoing and very dynamic process. There are multiple strategies for transforming natural macromolecules into sophisticated value-added products. Some methods include chemical modification of macromolecules, while others include blending several components into one new system. One of the most promising approaches for incorporating renewable macromolecules into new products is the synthesis of hybrid networks based on one or more natural components. Each one has unique characteristics, so its incorporation into a network brings new sustainable materials with properties that can be tuned according to their end-use. This article reviews the current state-of-the-art and future potential of renewable natural macromolecules as sustainable building blocks for the synthesis and use of hybrid polymer networks. The most recent advancements and applications that involve polymers, such as cellulose, chitin, alginic acid, gellan gum, lignin, and their derivatives, are discussed.

## 1. Introduction

**Figure 1:**
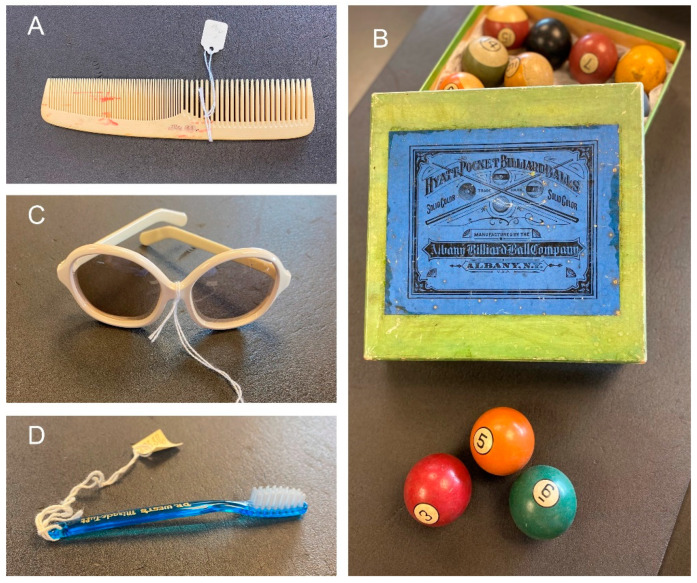
(**A**) Dupont comb, cellulose nitrate; (**B**) Billiard balls in original packaging, cellulose nitrate-based composite; (**C**) Sunglasses, cellulose acetate; (**D**) Dr. West’s miracle-tuft toothbrush, cellulose acetate and nylon. Plastics Artifact Collection, Special Collections Research Center, Syracuse University Libraries.

Humanity has been using natural polymers since day one of civilization. Cotton plant cultivation (~90% cellulose, a polysaccharide) has been verifiably practiced for 7800 years, with 6000-year-old cotton fabrics found during archaeological excavations in Peru [1]. Wool (a protein mixture with low lipid content) appeared as clothing material around the same time [2]. Wood (a composite of natural macromolecules cellulose, chemi-cellulose, and lignin) was used to make tools and provide shelter. For millennia, these natural materials have been exploited to cover various needs, and naturally, with the increase in population and the need for more reliable and stronger tools, humans started to explore ways of modifying and improving what was easily available from nature. The onset of the deliberate modification of those renewable natural polymers and macromolecules can be traced to the mid-19th century with the invention of cellulose nitrate, made by treating cotton with nitric and sulfuric acids [3]. Regrettably, the first envisioned use of the new material was for military purposes as “Schiesswolle” (gun cotton) [4]. Further adjustments to the method yielded a new material called “celluloid” (CLD), a colloidal mixture of nitrocellulose and camphor. This invention is mostly associated with the name of John Wesley Hyatt, who introduced it for numerous everyday uses, from hair combs (Figure 1A) to billiard balls (Figure 1B), collars, cuffs, and shirtfronts [5]. CLD was also the mainstay of the film industry until the 1920s, when it was replaced with cellulose acetate (CA) as the film base. First prepared in 1865, CA turned out to be a commercial success and played an important role in material manufacturing for cinematographic and photographic films, frames for eyeglasses (Figure 1C), packaging containers, and more. CA was also combined with other polymers. An example is shown in Figure 1D.

The petrochemical revolution in the 20th century introduced new precursors for polymers that led to the creation of a wide variety of oil-based materials. By the year 1930, practically every branch of human activity took advantage of the remarkably versatile properties and longevity of synthetic polymers [6]. However, with the gradual depletion of nonrenewable resources and environmental concerns caused by increased non-biodegradable waste, the research focus turned again to renewable biomass resources for the development of hybrid materials [7,8]. These raw materials are beneficial due to the short period of their replenishment cycle, which leads to the continuous flow of production supplies. Renewable feedstock resources include a wide variety of materials, such as carbon fibers, clay, plant-based oils, lignin, vanillin, tannin-based materials, and even pineapple leaf fibers [9]. Hybrid materials combine natural and synthetic macromolecules into one system, making up a rapidly emerging structural class in material science [10]. These systems possess properties superior to the sum of their components. One particularly intriguing group of hybrid materials is physically or chemically cross-linked gels and hydrogels. They have been extensively studied and used in diverse applications [11]. Many of them contain natural polymers with functional groups along their backbone suitable for multi-molecular assembly. While the combinations of natural and wholly synthetic network components are numerous, this review will focus only on those that involve polysaccharides (cellulose, chitin, alginic acid, gellan gum) and polyphenols (lignin).

## 2. Networks: Classification and Applications

**Figure 2:**
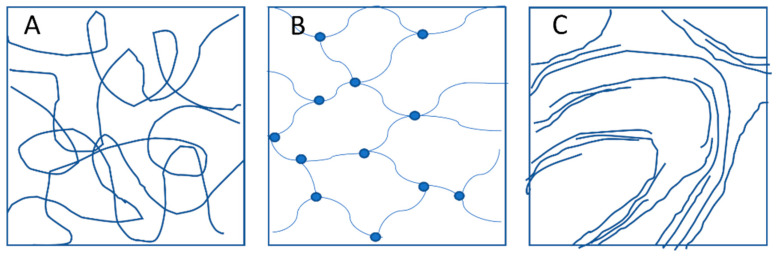
Classification of networks. (**A**) Entanglement networks; (**B**) Covalently cross-linked networks; (**C**) Physical gels with multiple chain junction zones.

Networks are complex systems classified into three main groups: “entanglement networks” (Figure 2A), chemically cross-linked networks (Figure 2B), and physical gels (Figure 2C). In the case of carbohydrates, entanglement networks are also often considered physical gels since, when dissolved, they actively participate in various interactions, such as hydrogen bonding and static interactions. This process is commonly called gelation and is described for various biopolymers, such as gelatin [12], gellan gum [13], alginic acid [14], and starch [15]. Chemically cross-linked networks are multi-molecular systems where polymer chains are covalently bound via a cross-linker [16]. In physical gels, cross-links do not form through chemical bonds on the polymers’ backbone but occur by so-called “junction zones”—a lateral aggregation of chains [17]. 

**Figure 3:**
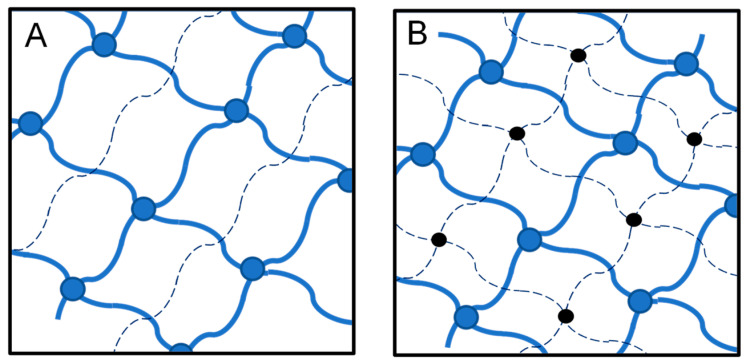
Schematic representation of SIPNs (**A**) and IPNs (**B**).

Gels based on biopolymers are typically hydrophilic and, therefore, can bind and retain large quantities of water without dissolving. Such gels are called hydrogels, and in general, they consist of cross-linked polymers with 3-dimensional (3D) network structures. It is known that the cross-linking method affects the morphology of hydrogels and their physicochemical properties [18]. Physical hydrogels are reversible and tend to respond to stimuli rapidly, but they are often fragile and sensitive to the chemical environment. Covalently cross-linked hydrogels, on the other hand, are solid and stable, but they are more rigid and cannot rapidly respond to stimuli due to their highly cross-linked structure. Therefore, the type and conditions of synthesis must match the envisioned application of the resulting material [19]. Networks can be synthesized via various strategies and methods. When utilizing a natural macromolecule, one must take into consideration its solubility, reactivity, and processability. If any of these properties are not suitable for the method chosen, the macromolecule must undergo a chemical modification or physical treatment. The deliberate combination of the above-described cross-linked systems produces new types of polymer gels labeled as semi-interpenetrating polymer networks (SIPNs) and interpenetrating polymer networks (IPNs) [20]. In SIPNs, the first polymer is cross-linked (blue dots in Figure 3A), and the second polymer (doted lines in Figure 3A) is linear and non-crosslinked; therefore, it can be extracted and washed off [21,22]. Within the IPN, one network consists of chains of polymer 1 cross-linked with cross-link 1 (blue dots in Figure 3B). Separately, chain 2 is cross-linked with cross-link 2, shown as black dots in Figure 3B. An important characteristic of the IPN is the absence of a covalent bond between the two distinct networks [23]. Depending on the type of polymers that form the IPN or SIPN, their applications vary and range from industrial and military [24,25] uses (sound and vibrational damping materials [26,27], shape-memory foams [28]) to biomedical uses [29], such as tissue engineering [30,31], drug delivery [32], wound dressing [33], and even dentistry [34].

Natural polymers are typically limited in their reactivity and processability, and corresponding physical gels usually lack mechanical strength and thermal stability. That is why their inclusion into IPNs is often the best method to overcome those limitations, as IPNs possess reduced interfacial tension and increased adhesion between the phases that lead to more stable morphology [35]. Such new hybrid materials have advantageous chemical, physical, and biological properties. Among the most widely used sustainable IPN and SIPN building blocks are carbohydrates, such as cellulose in its various forms and states, chitin as a cellulose analog, alginic acid, and gellan gum. The second most abundant natural macromolecule, lignin (a polyphenol), is extensively incorporated, as well. This review describes hybrid networks (entanglement networks, covalently cross-linked networks, and physical gels) containing those natural polymers and the recently reported synthetic routes for achieving the synthesis of various sophisticated hybrid networks.

## 3. Cellulose-Based Networks

**Figure 4:**
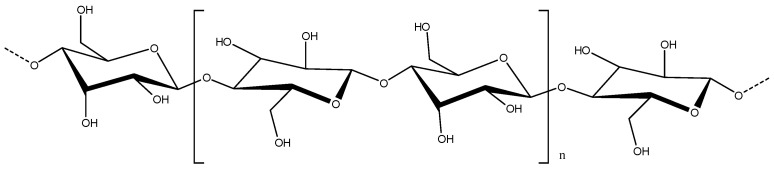
Structural formula of the β-l,4-glucan polymer chain. Cellobiose is shown in brackets. n—degree of polymerization.

Cellulose is the most abundant polymer on Earth and is considered one of the most important structural elements in plants and other living species. Each day a tree produces about 10 g of cellulose, making the global annual production of cellulose 1.5 × 10^12^ tons [36]. Cellulose is an unbranched polymer-homopolysaccharide composed of β-1,4-linked glucose residues. It is linear, and every other glucose unit is rotated approximately 180° with respect to its neighbors, giving cellulose a 2-fold axis. The physical properties such as crystalline state, degree of crystallinity, and molecular weight may be highly variable and depend on the source from which it was obtained [37]. Figure 4 shows the structural formula for the β-l,4-glucan polymer chain (cellulose). The repeating unit is called cellobiose and has a length of 1.03 nm. Its n—degree of polymerization—can reach up to 15 k in natural cellulose.

The biggest limitation of using cellulose in organic reactions is its solubility. Typically, to create an entanglement network, cellulose must be dissolved completely to ensure its homogeneity in the system. Its dissolution is quite challenging due to the partial crystallinity and a high degree of polymerization (DP). Cellulose is not soluble in water, nor in the majority of organic solvents, so multiple other systems are used, such as ionic liquids, LiOH/urea, NaOH/urea, NaOH/thiourea, and others [38].

**Figure 5:**
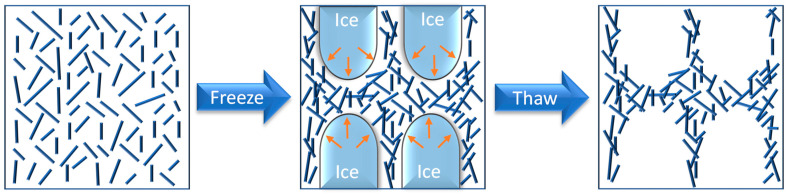
Preparation of physical gels of cellulose nanofibers using the freeze–thaw (FT) method.

Physical gels of cellulose can be created using the freeze–thaw (FT) method, where growing ice crystals upon freezing create the irreversible aggregation of physically confined cellulose nanocrystals between ice domains, and upon thawing, a network of aggregated cellulose fibers is formed (Figure 5). The described FT procedure yields a gel in liquid nitrogen at relatively low concentrations (~4 wt%) either in water or in polar organic solvents, producing hydrogels and organogels, respectively [39]. The FT method works for carboxymethylcellulose (CMC), as well. CMC is readily soluble in water, so to decrease the solubility, the ionized carboxyl groups must be protonated. Treatment with sulfuric acid yields insoluble and flexible transparent CMC-based films, allowing for the creation of a water-impermeable layer. When adhered to a filter paper, the hydrogels kept the surface on top of the paper waterproof for more than 24 h [40]. When an aqueous solution of citric acid was used, edible, highly biodegradable hydrogels with high compressive recoverability could be prepared [41].

Covalently cross-linked networks based on unsubstituted cellulose can be prepared when cross-linked via epichlorohydrin [42] or N,N′-methylene bisacrylamide [43]. An interesting case of a co-polymerized cross-linked hydrogel of microcrystalline cellulose and polyvinyl alcohol was reported by combining both physical and chemical cross-linking methods. This hydrogel was tested for the loading and release of a 5-fluorouracil drug in phosphate-buffered saline [44].

**Figure 6:**
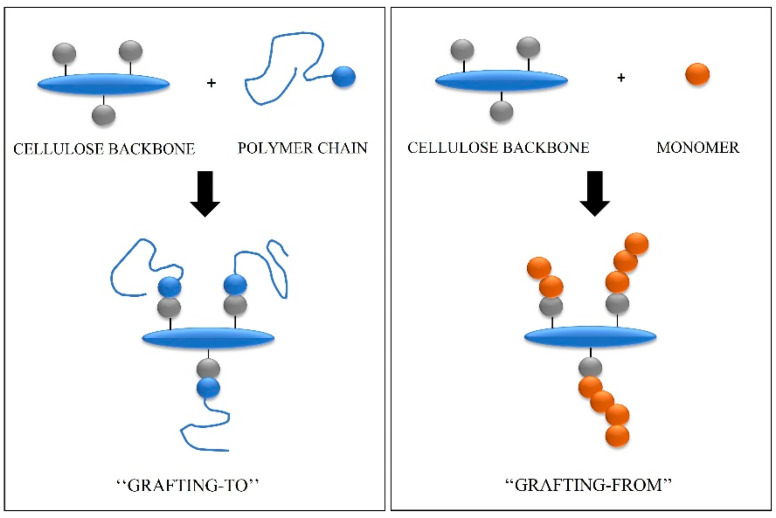
Grafting approaches used to modify carbohydrate’s backbone.

Chemical modification of cellulose fibers seems to be the most common approach to cellulose integration into gels and composites. Altering the surface of cellulose fibers helps to change the hydrophobicity, reactivity, and solubility of chains [45]. There are multiple ways to chemically modify the surface of cellulose, but grafting methods remain the most common. Figure 6 represents the two most frequently used approaches, called “grafting to” and “grafting from”. These methods are employed to modify the backbone of other carbohydrates as well.

**Figure 7:**
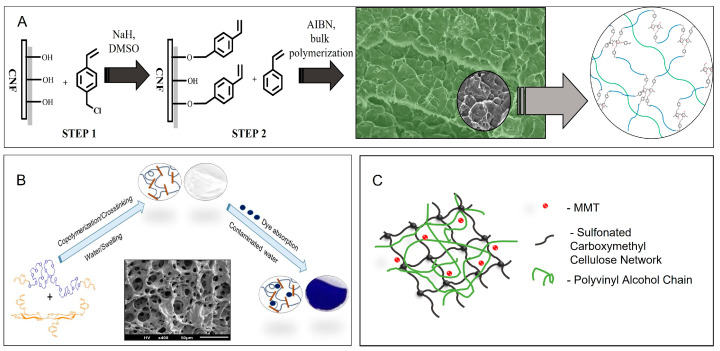
Various cellulose-based networks. (**A**) SIPN prepared after copolymerization of cellulose macromonomers (“cellu-mers”) and styrene [46]; (**B**) Hydrogel based on cellu-mers and modified PEG [47]; (**C**) Carboxymethyl cellulose-based SIPN nanocomposite [48].

Grafting can also produce reactive cellulose-based macromonomers. The reaction between the hydroxyl groups on the surface of cellulose fibers and 4-vinylbenzyl chloride enables the formation of cellulose-based cross-linkers, called “cellu-mers”. They can undergo radical copolymerization with either styrene to form SIPNs (Figure 7A) [46]. This strategy allows for the synthesis of versatile homogeneous sustainable networks, where incorporated cellulose fibers do not agglomerate, and synthesized materials have improved mechanical and thermal properties. The method was further extended with modified poly(ethylene glycol), PEG, to form hydrogels in high yields (Figure 7B) [47]. The PEG/cellu-mer hydrogels were able to bind and remove several organic dyes from an aqueous solution, showing potential for water remediation. Olad et al. described the synthesis of an amphiphilic SIPN nanocomposite where sulfonated carboxymethyl cellulose-*g*-poly(acrylic acid-*co*-acrylamide)/polyvinyl alcohol/montmorillonite was synthesized using free-radical graft copolymerization in an aqueous solution (Figure 7C). The hydrogels showed excellent swelling ability, indicating their potential application in agriculture [48].

Some other examples include the preparation of a hydrogel based on quaternary cellulose via an amino-anhydride “click” reaction between maleic anhydride copolymer and poly(acrylamine hydrochloride) [49]. The synthesis was performed in a “green” solvent (water), and the SIPN was tested as a super adsorbent for wastewater remediation purposes. Another strategy to synthesize cellulose-based IPN was reported, where doubly cross-linked graphene oxide modified cellulose nanocrystal/poly(N-isopropylacrylamide) IPN was prepared using an ultrasound one-pot strategy. The material was able to adsorb and remove organic dyes such as Congo red (CR) and methylene blue (MB) from the solution. They showed excellent water-swelling properties and good mechanical properties, making them a low-cost material for the purification of dye wastewater [50].

These research reports, selected among many, prove the importance and continuous development of cellulose-based networks and gels via multiple strategies and approaches.

## 4. Chitin-Containing Networks

**Figure 8:**
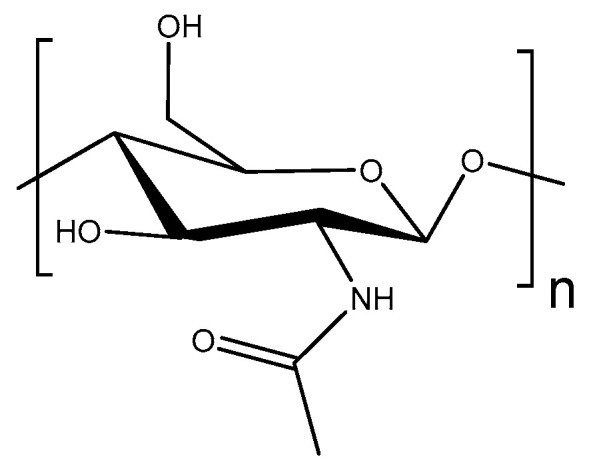
Chitin repeating unit. n—degree of polymerization.

Chitin is the second most abundant polysaccharide in nature after cellulose and is considered analogous to it as a structural material [51]. It is usually obtained by extraction from arthropods, nematodes, and fungi. The polymer is insoluble in common solvents and cannot be extracted by a solvent extraction method, which makes the process tough and laborious. Chitin tends to form microfibrils (also referred to as rods or crystallites) of around 3 nm in diameter that are stabilized by H-bonds involving the N-glucoacetate residue and hydroxyl groups (Figure 8). This acetamide group in its structural repeating unit makes the H-bonding in chitin even more complex and strong than that in cellulose.

**Figure 9:**
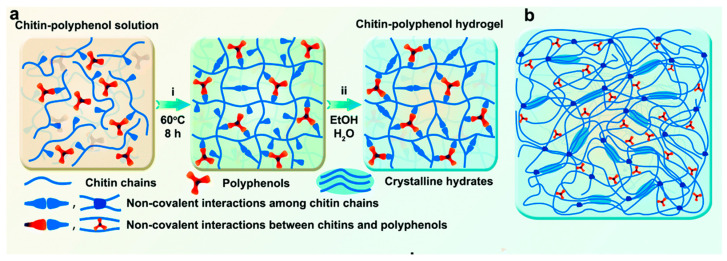
Preparation of the chitin–polyphenol hydrogel. (**a**) Processing principles; (i) Partial noncovalent network; (ii) Final formation of a multiple noncovalent network; (**b**) Chitin–polyphenol hydrogel. Modified with permission from [56]. Copyright 2023, published by Royal Society of Chemistry.

Due to its limited solubility in most organic solvents, the use of chitin is significantly limited to a few applications [52]. Ionic liquids are used to dissolve chitin and prepare relatively weak gels, but they still require high concentrations to form a gel [53]. Ionic liquids such as 1-butyl-3-methylimidazole acetate (BmimAc) dissolves native chitin and, upon regeneration, form a gel-like substance [54]. There are multiple other examples of physical gel formation by dissolving chitin from different sources in ionic liquids [55]. One of them is a preparation of a tannic acid-mediated self-assembled chitin hydrogel that has improved strength (8.18 MPa) and Young’s modulus (0.36 MPa) compared to pure chitin hydrogel. Due to the high noncovalent cross-linking density, the hydrogel enabled effective energy dissipation and high toughness, Figure 9 [56].

**Figure 10:**
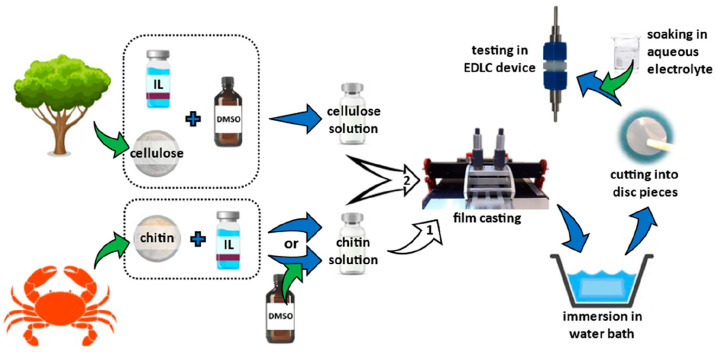
(**A**) The preparation of hydrogel electrolytes. Modified with permission from [57]. Copyright 2023, published by Springer Nature.

Kasprzak and Galiński have recently reported the synthesis of a chitin-based electrolyte with a solution-casting technique using ionic liquids. This method allowed the creation of highly efficient hydrogels in terms of specific capacitance, power density, and cyclability. In addition, this approach can also be used to synthesize chitin/cellulose-based hydrogel electrolytes. Specific capacitance values were in the range of 92–98 F·g^−1^, with excellent capacitance retention (97–98%) after 20,000 galvanostatic charge and discharge cycles. Such results show that chitin is a promising component to be used in the electrochemical industry to produce green electrochemical capacitors, Figure 10 [57].

**Figure 11:**
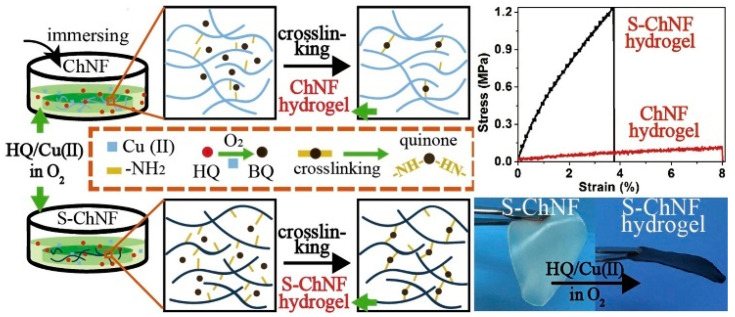
Comparison of chitin–based hydrogels prepared with chitin nanofibers (ChNFs) and surface–deacetylated chitin nanofibers (S-ChNF). Reproduced with permission from [59]. Copyright 2023, published by Elsevier.

Natural chitin–polyphenol hydrogels were reported to be highly biocompatible and biodegradable, making them promising materials for future tissue engineering [58]. Typically, chitin-based physical gels have low gel stability with weak strength, and an irreversible covalent cross-linking alleviates the above-mentioned problems. In 2019, Chen et al. reported the synthesis of hydrogels based on quinone-crosslinked chitin nanofibers using amino groups. There, surface-deacetylated chitin nanofibers (S-ChNF) formed a hydrogel after a reaction in hydroquinone (HQ)/copper (Cu(II)) solutions. Compared to non-surface-deacetylated, S-ChNF-based hydrogel displayed almost 10-fold higher tensile strength because of the extended cross-linking effect between quinone and amino groups, Figure 11 [59].

**Figure 12:**
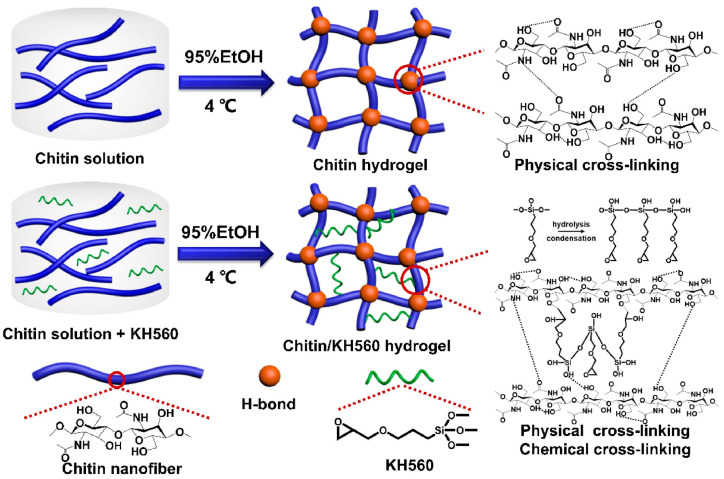
Preparation of double cross-linked chitin/KH560 hydrogel. Reproduced with permission from [60]. Copyright 2023, published by Elsevier.

Other examples of recently reported chitin-based gels are the double cross-linked chitin/KH560 networks [60]. Such systems have two types of cross-links: physical and chemical. They can be synthesized when dissolving chitin in an aqueous solution of KOH/urea with a freeze–thaw process followed by using KH560 (an epoxy-functional silane) as a cross-linking agent and coagulating in ethanol solution at low temperature. The material was biocompatible, with excellent mechanical properties and a high swelling ratio. The presence of two types of cross-links makes this hydrogel an excellent damping material since cross-linking bonds can serve as “sacrificial bonds”, effectively dispersing the stress and withstanding large deformations, Figure 12.

A similar approach was used for the synthesis of injectable thermosensitive hydrogels. Double cross-linked furyl-modified hydroxypropyl chitin polymer was synthesized homogeneously in an aqueous solution. Firstly, physically cross-linked hydrogel based on furyl-modified hydroxypropyl chitin was formed upon cooling to 4 °C. Then, irreversible cross-linking with maleimide-terminated PEG 2k Da using the Diels–Alder reaction was achieved under physiological conditions. Potentially, during the second step, there was sufficient time to stir-in various therapeutic molecules and/or cells, making these gels an excellent material for drug delivery, three-dimensional cell culture, and tissue repair. Additionally, dually cross-linked hydrogels have enhanced mechanical strength compared to physically cross-linked ones [61].

A SIPN hydrogel could also be prepared via radical polymerization. Unsaturated poly(vinyl alcohol)/maleic anhydride-based macromonomer was cross-linked with acrylic acid in the presence of water-soluble chitin to generate a SIPN. Linear chains of chitin were homogeneously dispersed throughout the network. Such a completely sustainable synthetic method allowed for the preparation of non-toxic, biocompatible, and robust hydrogels [62]. Along with other natural polymers, chitin composite hydrogels were evaluated for the effective adsorptive removal of textile dyes from water [63,64].

**Figure 13:**
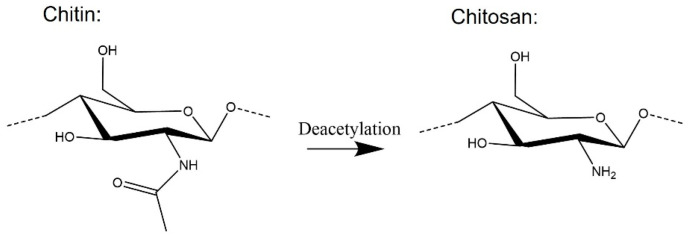
Transformation of Chitin to Chitosan.

Chemical or enzymatic deacetylation of chitin leads to the creation of a new biopolymer—chitosan, Figure 13 [65]. When the degree of deacetylation (DA) is higher than 50 mol%, the product is soluble in dilute acid solutions. This approach is frequently used to create advanced products based on chitin and chitosan. Their biological activity and antimicrobial properties make them perfect candidates for various applications, such as cell culture media [66] and antimicrobial materials [67].

**Figure 14:**
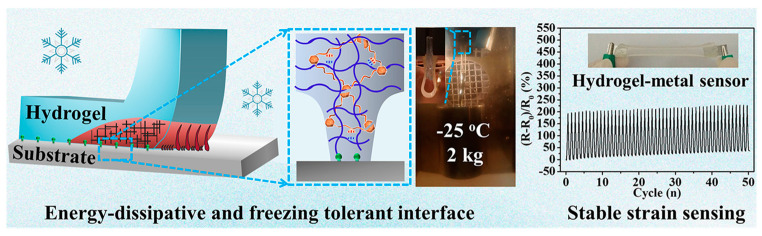
Chitosan–polyacrylamide double-network ionic hydrogel for flexible electronics. Modified with permission from [68]. Copyright 2023, published by Elsevier.

Networks based on chitosan are being investigated for various other purposes. Yang et al. in 2023 described the development of a robust, anti-freezing, and conductive double-network for a stable-performance flexible electronic device. Various chitosan–polyacrylamide double-network ionic hydrogels were chemically cross-linked with different substrates. The chitosan physical network enabled effective energy dissipation, while the covalent linkage between the polyacrylamide network and the substrate surface provided interfacial adhesion. The hydrogel–substrate combinations were reported to maintain high interfacial toughness at low temperatures. In addition, the high conductivity of the hydrogel–metal interface makes it a promising material for developing a flexible sensor to detect strain and pressure within a broad temperature range, Figure 14 [68].

Several other interesting examples of sophisticated chitosan-based systems were recently reported. Wan et al. reported a synthesis of poly(acrylic acid)/chitosan-*g*-poly(vinylamine) composite hydrogel for efficient adsorption of methylene blue dye from aqueous solutions. It was reported that the hydrogel achieved 85.24% removal of dye even after five adsorption cycles [69]. Another report announced the synthesis of chitosan-based hydrogel containing in situ bio-reduced silver nanoparticles for accelerated healing of infected full-thickness skin defects. This material could enable a ground-breaking sustainable anti-infection, anti-inflammation, collagen-stimulating treatment that promotes the formation of epithelia and blood vessels [70].

## 5. Alginic Acid Networks

**Figure 15:**
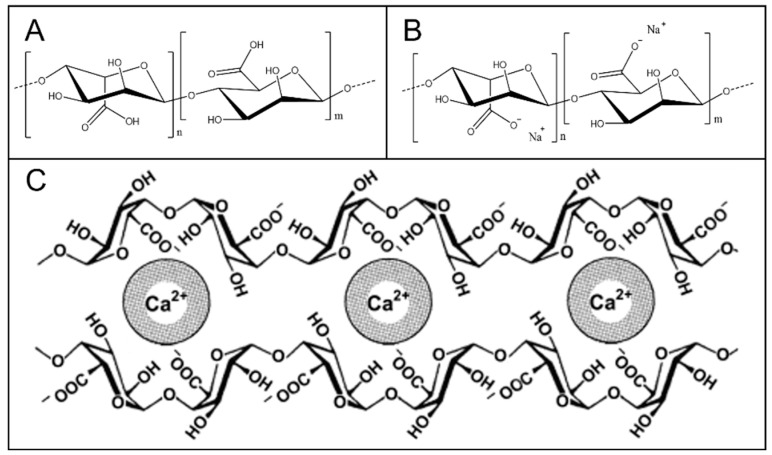
(**A**) Structural formula of alginic acid; (**B**) Structural formula of sodium alginate; (**C**) The “egg–box” model for alginate gelation with calcium ions. Ca^2+^ ions bind to the carboxyl groups of the guluronic acid residues of two neighboring alginate chains. Reproduced with permission from [74]. Copyright 2023, published by John Wiley and Sons.

Alginic acid (AA) is a natural polysaccharide produced by brown seaweeds such as kelp and macroalgae or produced by microbial fermentation using special bacteria [71]. Alginate consists of two basic building blocks, α-l-guluronic acid (G) and β-d-mannuronic acid (M) residues, linearly linked together with 1–4 linkages (Figure 15A). The composition and distribution of these M/G residues impact the properties and functionalities of alginate. Due to the nontoxicity and biocompatibility of this natural polymer, the most common use of AA-based networks is in biomedical, agricultural, and food-packaging industries [72]. Sodium alginate (SA) is the sodium salt form of alginic acid, frequently used for the synthesis of gels and networks (Figure 15B).

The “egg-box” model is a classic model describing the gelation mechanism of alginates. The most recent studies show that the chains pair into dimers cooperatively when the ratio of Ca^2+^ ions to G units in alginate R_(Ca/G)_ is 0.25 for higher G alginate and 0.50 for lower G alginate. At higher R, the “egg-box” dimers act as a structural basis for further lateral aggregation by successively doubling dimers into tetramers, octamers, and hexadecamers (Figure 15C) [73,74].

“Entanglement networks” based on alginates can be formed at lower pH when the chains associate. However, the gel is weak, soft, and soluble in alkaline solutions. Therefore, it does not find much practical use. Casting aqueous AA solutions leads to the creation of transparent and flexible films, but these films are water-soluble unless they are immersed in a multivalent ion salt solution [75]. In the presence of divalent cations like calcium (Ca^2+^) and magnesium (Mg^2+^), alginate chains cross-link and form water-insoluble physical gels [76]. Most recent reports include various other metals, such as strontium (Sr^2+^), zinc (Zn^2+^), and even aluminum (Al^3+^). Depending on the metal, hydrogels exhibit different properties that lead to various applications [77], but the drawback of the use of these materials is their low dimensional stability. Pure alginate gels formed with calcium ions have high porosity and are known for their healing potential and prospects in anti-tumor applications. However, they are unstable at physiological pH or in environments with a high concentration of phosphate or citrate ions because Ca^2+^ ions can be extracted from the hydrogel structures, leading to their collapse [78].

Covalently cross-linked alginate gels are more mechanically stable compared to physically cross-linked ones [79,80]. If the material is intended to be applied for biomedical purposes, biocompatible cross-linkers should be used. For example, glutaraldehyde was utilized as a cross-linking agent in the preparation of sodium alginate/poly(vinyl alcohol) IPN [81]. Although tests have indicated that the prepared IPN hydrogel membranes were safe for drug delivery applications, some concerns related to glutaraldehyde cytotoxicity remain, as it is a contact irritant and dermal sensitizer [82].

**Figure 16:**
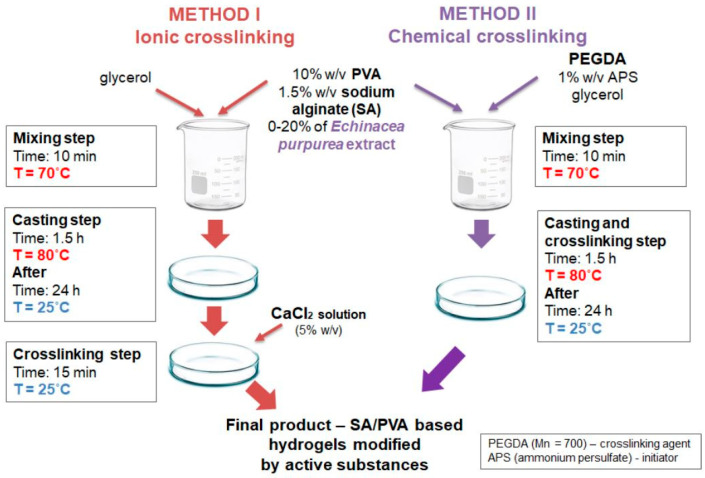
Preparation of physically– (Method 1) and chemically (Method 2) crosslinked gels. Reproduced with permission from [18]. Copyright 2023, published by MDPI.

Biocompatible chemically cross-linked gels based on modified sodium alginate/poly(vinyl alcohol) using poly(ethylene glycol) diacrylate as a cross-linking agent were recently reported. The authors have compared the mechanical strength of chemically cross-linked hydrogels with those prepared via a physical cross-linking mechanism using Ca^2+^ (Figure 16) [18]. They also studied the drug release profile using *Echinacea purpurea* (*EP*) extract as a model compound. Results indicated that hydrogels prepared via ionic cross-linking had better swelling than those prepared via chemical cross-linking. However, chemically cross-linked ones had enhanced mechanical strength, and the surface of the hydrogels was more regular and denser than in physical gels. Analysis of release profiles of the model drug showed that *EP* could be delivered in a controlled manner for an extended time, but only with the alginate-based hydrogels synthesized using chemical cross-linking. In the case of physical gels, an undesirable phenomenon called a “burst effect” was observed, which is a very rapid release of the active substance due to rapid surface desorption instead of slow and steady release.

Chemically cross-linked alginate hydrogels were reported to recover gold nanoparticles from wastewater effectively. Particles of sericin and alginate chemically cross-linked by proanthocyanidins were synthesized and characterized. Sericin is usually discharged in wastewater as a silk manufacturing by-product; therefore, the synthesized hydrogel is interesting not only as a system able to recover gold from aqueous media but also as a possible solution to problems with sericin disposal [83].

Kim et al. proved that the formation of IPN or SIPN not only improves the mechanical and thermal stability of alginate-based gels, but this improvement happens without losing the advantages of polysaccharides. A series of SPNs were prepared with 75:25, 50:50, and 25:75 ratios of alginate to poly(vinyl) alcohol, where there is only one cross-linked network inside—alginate. Results revealed that by utilizing the same components but altering the method of hydrogel preparation, pH-responsive behavior, stability, and water retention properties can be controlled and altered according to the desired uses [19].

**Figure 17:**
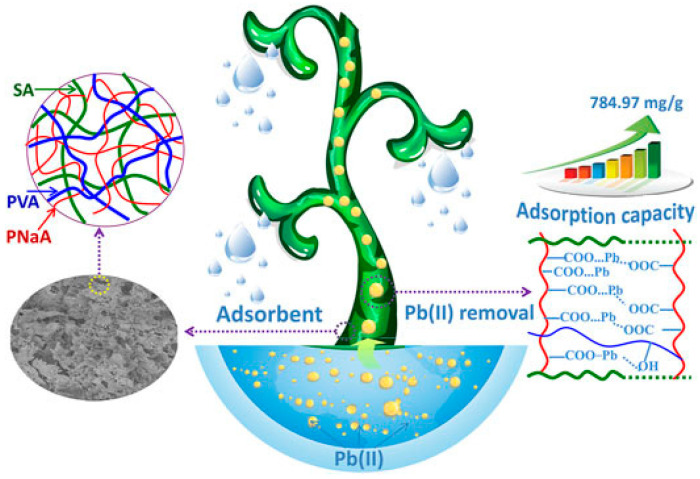
Structure of the network and its adsorption actions for Pb(II). Reproduced with permission from [84]. Copyright 2023, published by Frontiers in Chemistry.

Alginate IPNs and SIPNs can be utilized for other interesting applications. For example, Wang et al. synthesized reusable porous alginate/poly(vinyl alcohol) SIPN hydrogel to remove Pb(II) from aqueous solutions [84]. They used a natural biopolymer sodium alginate (SA) as the main chain, sodium acrylate (NaA) as the monomer, and poly(vinyl alcohol) (PVA). NaA monomers were grafted onto the SA chains, while PVA chains were interpenetrated and entangled within the cross-linked network. This facile grafting polymerization in an aqueous medium created hydrogels with excellent adsorption properties and removal efficiency toward Pb(II). Incorporated PVA polymer chains improved the regularity of the network and, therefore, increased the mechanical strength and hydrophilicity. More importantly, ~94% of the bound Pb(II) can be de-adsorbed and recycled using an acid-leaching process. The system can be regenerated five times, with the possibility to de-adsorb and recycle about 89.9% of the initially sequestered Pb(II). In addition, the adsorption capacity for Pb(II) could reach up to 502.5 mg/g after being reused for five cycles. Figure 17 shows the structure of the SIPN and its adsorption actions for Pb(II).

**Figure 18:**
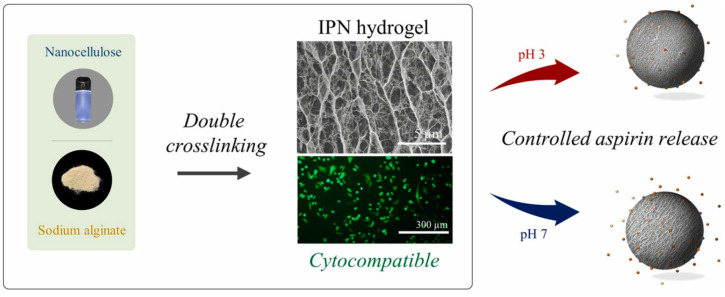
Synthesis of sodium alginate/nanocellulose-based IPN for controlled aspirin release. Reproduced with permission from [87]. Copyright 2023, published by Elsevier.

Notably, the most common use of alginate-based IPNs is for biomedical purposes. When they are used for controlled drug-release, the loaded substance can be retained to the maximum extent without negatively affecting its pharmacological effect due to the excellent biocompatibility and stable porous structure of the network [85,86]. For example, controlled delivery of aspirin using IPN hydrogels based on sodium alginate and nanocellulose was reported recently [87]. The first framework network made with sodium alginate and nanocellulose was built after acetic acid coagulation, followed by CaCl_2_ chelation. Ions of Ca^2+^ can exchange ions with sodium alginate and chelate with carboxyl groups on the surface of cellulose to form a uniform interpenetrating network structure. The resulting hydrogel showed good cytocompatibility and nontoxicity, was mechanically robust, and had a prolonged aspirin release time of ~ 80 h, making it an ideal system for prolonged aspirin release (Figure 18).

Alginate-based hydrogels have gained increased interest as an alternative to the existing clinical methods for treating cartilage injuries [88]. Injectable in situ forming hydrogels as carriers for drug delivery or scaffolds for tissue engineering applications offer multiple benefits as they require a minimally invasive procedure for implantation. The cross-linkers used for this application should be carefully selected, as they should show no cytotoxicity towards encapsulated cells and should provide an appropriate gelation time after the injection. An example of a suitable cross-linker was recently reported describing a synthesis of an injectable IPN based on modified cartilage extracellular matrix (ECM) and alginate [89]. Horseradish peroxidase (HRP) in the presence of hydrogen peroxide was used as a cross-linking mediator after the extracellular matrix was functionalized with phenol groups (ECM-ph). The synthesized injectable hydrogel was then impregnated with silk fibroin nanofibers (SFN). It was evaluated for articular cartilage tissue engineering applications. The material provided a suitable microenvironment for cell growth and viability. Collagen content measurements showed that this hydrogel has great potential and capability to mimic natural cartilage. Mechanical strength, however, needed further improvement [89].

## 6. Gellan Gum-Derived Networks

**Figure 19:**
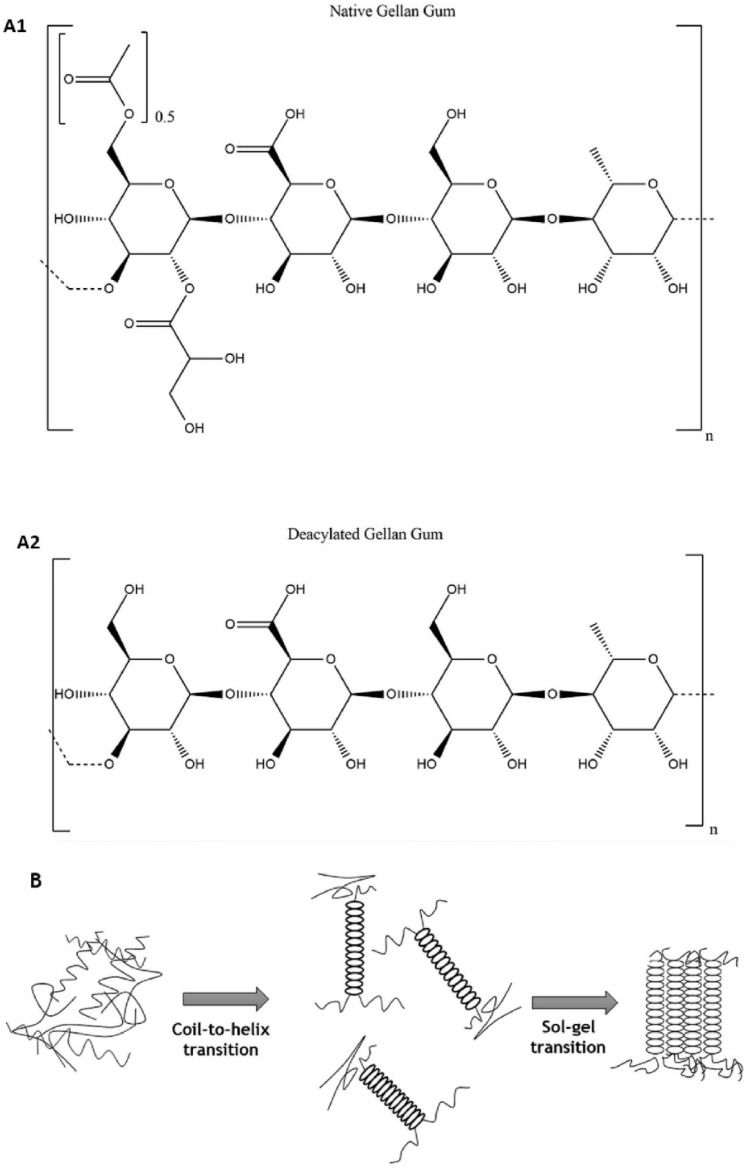
Chemical structure of high acyl (**A1**) and low acyl (**A2**) gellan gum; Gelation process of gellan gum (**B**). Reproduced with permission from [91]. Copyright 2023, published by Elsevier.

**Figure 20:**
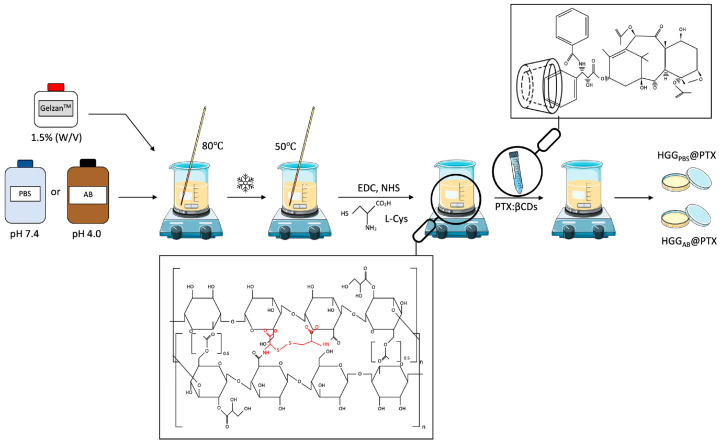
Preparation of GG-based hydrogels loaded with paclitaxel for local breast cancer therapy. Reproduced with permission from [97]. Copyright 2023, published by Elsevier.

Gellan Gum (GG) is a linear electronegative exopolysaccharide produced by *Pseudomonas elodea*. Its main chain consists of four repeating carbohydrate units: two D-glucose, one L-rhamnose, and one D-glucuronic acid (Figure 19A). On average, about 25% of repeating units contain an o-acetyl group linked to the C-6 position of one of the δ-D-glucopyranosyl units [90,91]. GG is in coil form at elevated temperatures in solution, but upon cooling, the coil transforms into a thermally-reversible double helix. These anti-parallel double helices self-assemble and form oriented bundles that are connected via untwined regions of polysaccharide chains (Figure 19B) [92]. GG is biodegradable and nonpathogenic (approved by the FDA) and allowed for use as a food additive [93]. However, it is investigated for other applications, as well [94]. The most investigated use of GG hydrogels for biomedical purposes is for drug delivery [95]. Physical hydrogels based on GG that can bind and release glucose can be easily prepared by dispersing the hydrocolloid powder in water at 90 °C. Recently, hydrogels based on high acyl (HA) and a modified version—low acyl (LA)—GG have been studied and compared [96]. HA GG has acetyl and glyceryl substitutions on the first glucose of the repeating unit at the O-2 and O-6 positions, while in LA, GG acyl groups are removed (Figure 19(A1,A2)). Synthesized HA hydrogels had a much greater swelling ratio than LA hydrogels, and both had more significant swelling in DI water than in 50 mM KCl solution. Swelling slows the release of glucose by decreasing the diffusion flux, which must be considered when developing GG-based hydrogels. However, the hydrogels were brittle; to improve the mechanical strength, chemical cross-linking must be considered as an alternative synthesis. An example of this approach was recently published. Covalently cross-linked redox-responsive implantable hydrogels were synthesized and used as paclitaxel carriers for HER2-positive breast cancer therapy [97]. These systems can be prepared using two types of buffers: acetate buffer and phosphate buffer, cross-linked with different concentrations of l-cysteine (Figure 20). The hydrogels were loaded with paclitaxel:β-cyclodextrin, and in vitro tests indicated that they expressed great anti-tumor activity. Rheological analysis showed that samples had characteristic strong gel behavior and could withstand the load over a broad range of frequencies. Potentially, they could be used in the post-surgical treatment of HER2-overexpressing breast tumors [97].

Three-dimensional (3D) printing is increasingly used to produce hydrogel structures with increased complexity and recoverable mechanical properties [98]. GG-based hydrogels are increasingly popular as novel biocompatible inks for printing, as evidenced by a recent review [99]. Here, only a few newly published articles will be discussed in detail.

**Figure 21:**
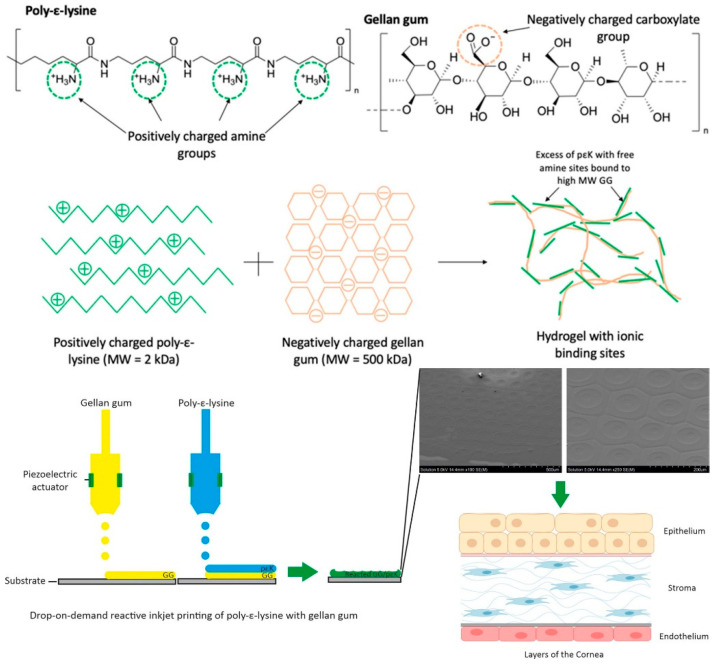
Chemical structures of poly(ε-lysine), gellan gum, their hydrogel, and reactive inkjet 3D printing. Reproduced with permission from [100]. Copyright 2023, published by Elsevier.

Duffy et al. reported using poly-ɛ-lysine/GG hydrogels for the 3D reactive inkjet printing of corneal constructs [100]. It is possible to modify the structure of the printed hydrogels to include pores if desired. The printed hydrogels were 80% transparent, cytocompatible with corneal epithelial and endothelial cells, and facilitated cell attachment to their surface (Figure 21).

**Figure 22:**
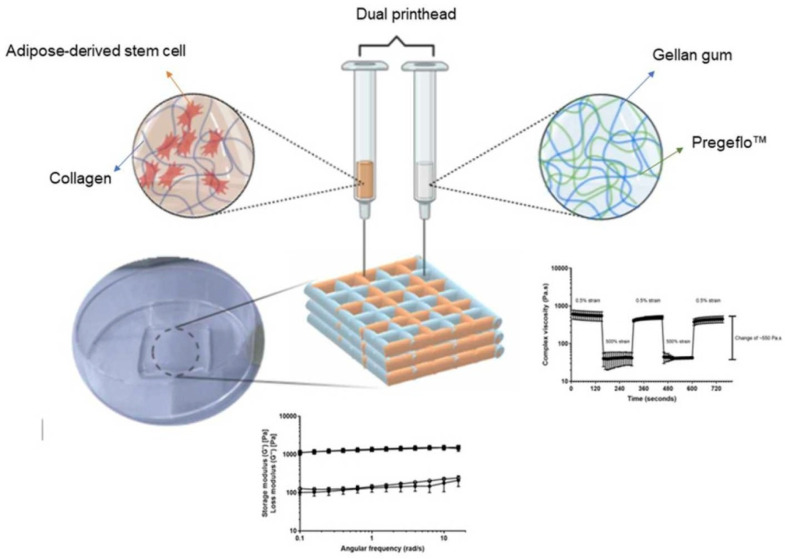
Schematic representation of printing the improved IPN hydrogel ink formula using a multi-printhead 3D bioprinter. Reproduced with permission from [101]. Copyright 2023, published by Elsevier.

Three-dimensional printing was also used to enhance the mechanical strength of the final IPNs product. For example, a starch-based excipient known as PREGEFLO^®^PI10 was used to reinforce a GG–collagen IPN hydrogel [101]. A geometric addition method was developed for a new biocompatible bio-ink by incorporating PREGEFLO^®^PI10 into the IPN loaded with adipose-derived stem cells. A multi-printhead pneumatic extrusion-based 3D bioprinter was used to bio-print the improved IPN ink to demonstrate the potential for on-site fabrication (Figure 22).

Environmental cleanup is another emerging field where GG-based IPNs were used. The failure to treat industrial waste before it is discharged into water streams is a significant problem for the industrial, agricultural, and health sectors. The removal of various organic dyes used industrially in food, pharmaceuticals, paper, and textiles is one of the most demanding water remediation tasks [102]. The presence of dyes in wastewater poses a great threat to living organisms as it prevents the passage of sunlight into the depths of water bodies, but also it is harmful to humans and animals who consume such water for their life needs [103,104].

**Figure 23:**
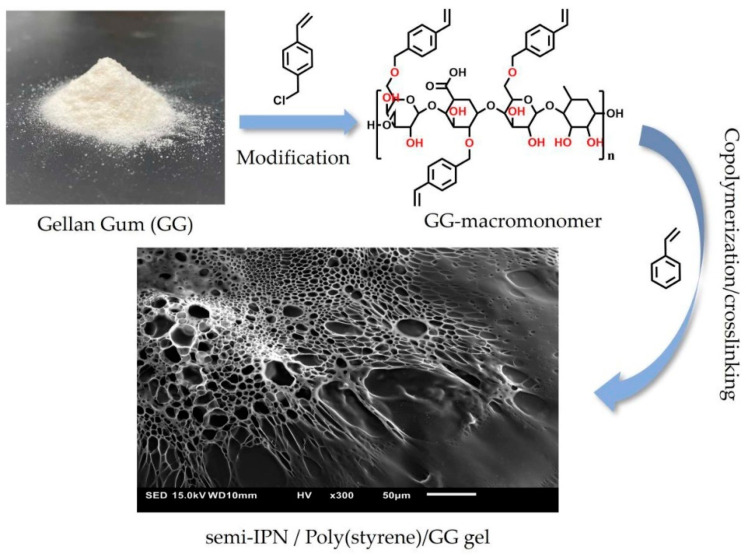
Synthesis of GG/PSt SIPN [106].

GG is a great material for wastewater remediation [105]. A multifunctional cross-linker with increased surface hydrophobicity was synthesized by grafting styrenic moieties onto the surface of GG [106]. These modified GG polymers could be co-polymerized with styrene, forming a SIPN network (Figure 23). These networks were mechanically robust, and their binding ability towards several organic dyes was high, showing that they could be used as potential materials in environmental cleanup [105].

## 7. Lignin-Based Networks

**Figure 24:**
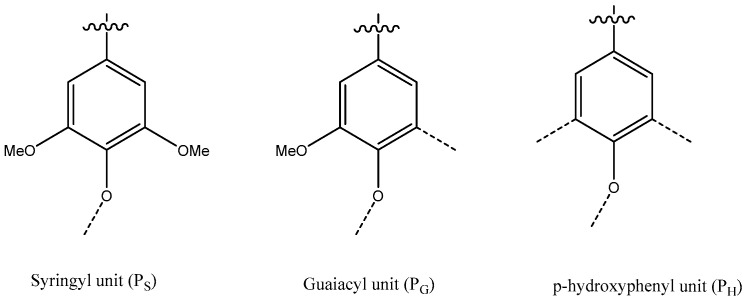
Lignin methoxyl substituted S-, G-, and H- moieties as generic P_S,_ P_G_, and P_H_ units. See also reference [110].

Lignin accounts for approximately 30% of all organic carbon and plays three main roles in plants: (a) it provides structural integrity to the cell wall and strength to the stem and root; (b) it waterproofs the cell wall to make the transport of water and solutes through the vascular system possible; (c) it protects plants against pathogens [107]. This complex, irregular, three-dimensional, amorphous, and interlinked biomacromolecule consists of phenylpropane units linked by C-C and C-O-C bonds. There are three monolignol moieties that exist as p-hydroxyphenyl (H), guaiacyl (G), and syringyl (S) units, Figure 24 [108]. Lignins in softwoods (SWs) are mainly composed of P_G_-units (with minor amounts of H-units), while hardwood (HW) lignins are mainly composed of G- and S-units. H-units’ content may be slightly higher in grasses [109].

**Figure 25:**
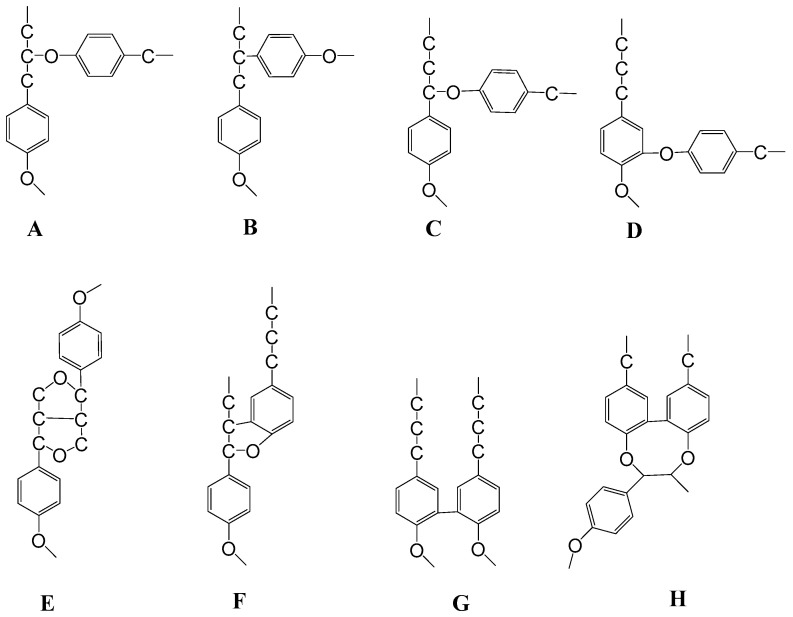
Various C–C and C–O–C bonds found in lignin structure. The main linkages are (**A**) β-aryl ether β–O–4; (**B**) diphenyl ethane β–1; (**C**) α-aryl ether α–O–4; (**D**) diphenylether 4–O–5 (from oligomer–oligomer couplings); (**E**) pinoresinol β–β; (**F**) phenylocumaran β–5 (from monomer–oligomer couplings); (**G**) biphenyl 5–5; (**H**) dibenzodioxicine 5–5.

The most frequent lignin linkage is the β–O–4-(β-aryl ether), and it is one of the most easily chemically cleaved (Figure 25A). The other linkages presented in that figure are more resistant to chemical degradation.

Lignin is the main by-product of the pulp and paper industry, and interest in its reutilization has dramatically increased. Two methods are generally used to repurpose technical lignin. The first one is the depolymerization of lignin, breaking it down into monomeric units with further modification to form functional materials. The second approach is a chemical modification of the existing polymer to obtain a functionalized polymer. The first method is more common since lignin has excellent potential as a renewable source of aromatic compounds in lieu of petroleum. Therefore, recently, many reports have related to the cleavage of the C–O and C–C linkages. For example, the most recent one is on the cleavage of C–C/C–O bonds in lignin using dye-sensitized photoelectrosynthetic solar cells. This method presents a promising direction for the sustainable production of chemicals based on lignin [111,112].

**Figure 26:**
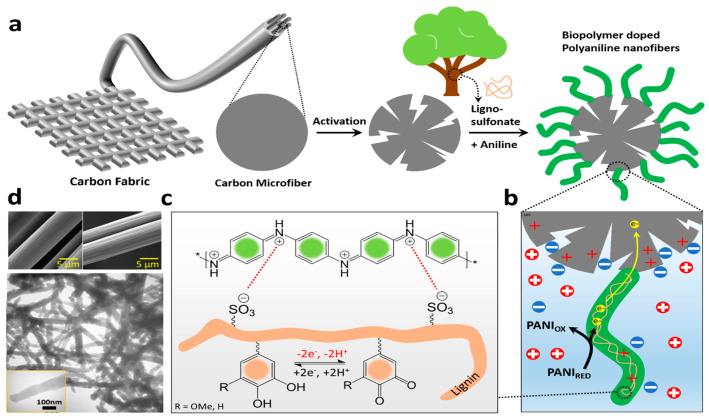
A supercapacitor energy storage device based on polyaniline and sulfonated lignin; (**a**) The preparation of biopolymer doped polyaniline nanofiber; (**b**) Faradaic charge transfer and electrical double layer charge accumulation at the electrode/electrolyte interface; (**c**) Structure and the interaction within the components in the nanocomposite; (**d**) SEM images of the carbon fiber substrate before (left) and after (right) electrochemical etching along with TEM image of the resulting PANI-LS nanocomposite. Reproduced with permission from [119]. Copyright 2023, published by American Chemical Society.

The incorporation of lignin in functional hydrogels is another direction for the upcycling of this natural macromolecule [113]. Physical networks are typically prepared by blending/mixing two or more polymers to immobilize lignin within the existing matrix of the polymer [114]. This leads to the creation of an IPN or a SIPN, where lignin provides mechanical strength, processability, and toughness to the network, just like it does for plants. These systems are being used for various applications, including but not limited to agriculture [115], wastewater treatment [116], biomedical [117], and energy storage fields [118]. A supercapacitor energy storage IPN device was produced using a one-pot electrosynthesis of polyaniline and sulfonated lignin [119]. The nanocomposite formulation was prepared on an electrochemically etched carbon fiber electrode (Figure 26). The constructed device displayed great energy/power performance and flexibility. This approach allows for the synthesis of metal-free electrode active materials from green and cost-effective sources and marks an essential step toward green energy technology.

**Figure 27:**
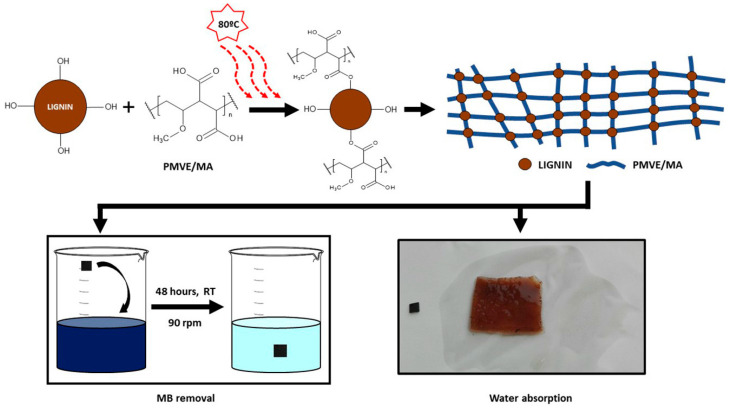
Proposed chemical reactions taking place during the cross-linking of different technical lignins with PMVE/MA via ester linkages. Reproduced with permission from [120]. Copyright 2023, published by Elsevier.

Lignin can also be used for environmental remediation purposes, for example, as “super-swelling” hydrogels for dye removal. In this case, technical lignins were cross-linked with poly(methyl vinyl ether-*co*-maleic acid), PMVE/MA, Figure 27 [120]. The results indicated that the adsorption of methylene blue from solutions was comparable to that reported for activated charcoals. An interesting and important fact is that these renewable and biocompatible hydrogels were prepared via a “green” approach, with no organic solvents or potentially toxic reagents used, and water was the main reaction by-product. This strategy produced a system that was not only prepared under conditions with minimal harm to the environment but was also able to remove contaminants from it [120].

**Figure 28:**
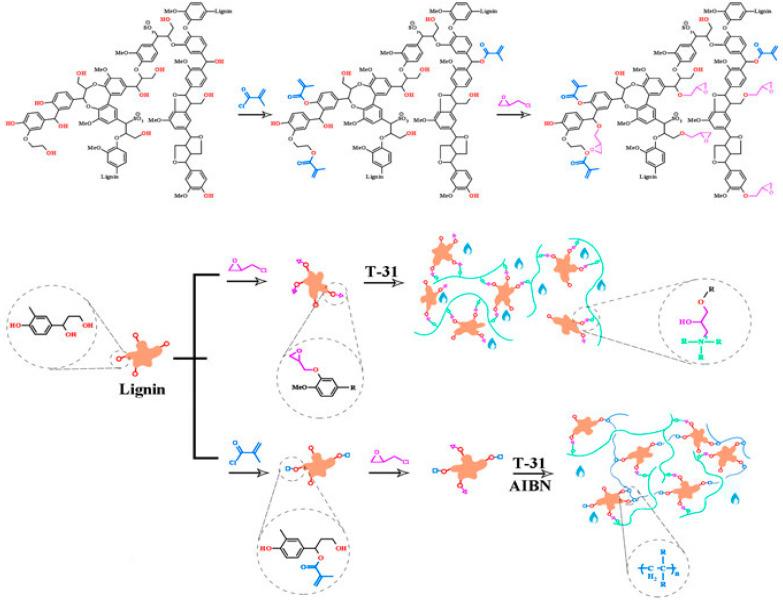
Schematic preparation of the double-IPN lignin-based epoxy resin adhesives resistant to extree environments. Reproduced with permission from [121]. Copyright 2023, published by American Chemical Society.

Multiple polar sites in lignin’s framework can be used for the physical cross-linking of hydrophilic polymers via H-bonding. Wang et al. utilized lignin in lieu of traditional bisphenol A to prepare lignin-based epoxy resin adhesives with double-interpenetrating network structures [121]. In their work, lignin was modified with methacryloyl chloride and epichlorohydrin, introducing double bonds and oxirane rings into the macromolecule to form the network (Figure 28). In that way, the free volume inside the gel can be reduced and prevent the infiltration of external water molecules. The final product could be used as a high-quality water-resistant adhesive.

## 8. Other Hybrid Networks

In recent decades, metal-coordinated networks (MOFs) and frameworks have been rapidly developing. MOFs are porous coordination polymers that have both organic and inorganic components. Metal cations such as Ag^+^, Zn^2+^, Co^2+^, Cu^2+^, and Mo^6+^ can be easily introduced to the frameworks, and their antibacterial activity is proven [122]. Metal–phenolic networks (MPNs), on the other hand, are non-toxic and more environmentally friendly due to the milder conditions of their synthesis. Polyphenols and metal ions readily dissociate at low pH, enabling the controlled release of a metal ion, with Fe being the most common metal. MPNs have great potential in chemotherapy, as they can produce highly toxic hydroxyl radicals (•OH) that effectively help the drug kill tumor cells [123].

The few examples discussed so far clearly indicate that work on sustainable hybrid networks is rapidly evolving and growing. Various renewable natural macromolecules can serve as sustainable building blocks for these materials. New complex systems are being developed and synthesized with properties designed and adjusted for specific applications. Reports that were not mentioned in the main text are listed in Table 1.

### Advantages of Hybrid Networks and Remaining Challenges

Renewable natural macromolecules used for the synthesis of hybrid polymer networks offer a range of advantages. They are non-toxic, biocompatible, biodegradable, and, more importantly, available all year round [135]. In addition, they vary in molecular mass, chemical structure, and reactive groups available. Those reactive groups often serve as helpful anchors for direct cross-linking, chemical modification, or chelation. Thus, natural macromolecules are good candidates to substitute or aid petroleum-based polymers, leading to minimizing the ecological footprint related to the extraction and processing of oil itself. Important future use might be in the biomedical field for vital cases such as tissue regeneration or the restoration of physiological functions [136].

There are, however, certain limitations related to the commercial use of natural polymers and their hybrid networks. A few of them are discussed here. The first problem is the lack of mass production due to modest efficiency in extraction and purification. This increases the final price of the natural polymers and macromolecules, making the materials based on them not economically practical in several key markets [137]. To alleviate this problem, many countries are trying to implement environmental laws that aim to increase mass production and use of sustainable raw materials [138]. The second problem stems from limitations in compatibility with synthetic polymers [139] and a lack of stability, reactivity, and processability [21]. Chemical modification is one of the most effective methods to modify the surface properties of natural materials and make them more compatible and disperse better within other polymers [140,141,142]. However, on an industrial scale, such modifications might not be cost-sustainable. Not only the modification agents can be harmful, but the solvents, as well. The third problem is associated with the characterization of the natural polymers and macromolecules due to the great dispersity in molecular mass, diversity in repeating units, and solubility before and after modification [143]. Nuclear Magnetic Resonance (NMR) is a powerful spectroscopic technique for the structure elucidation of small and large molecules but has certain limitations when used to characterize polysaccharides due to their poor solubility in most common NMR solvents and signal overlap from structurally similar moieties and fragments. That is why two- and three-dimensional techniques are increasingly used [144]. Since NMR is a colligative method, it cannot differentiate between macromolecules with different degrees of modification and shows merely the total content of newly incorporated reactive functionalities. Matrix-assisted laser desorption and ionization time-of-flight (MALDI-TOF) is a precise non-destructive mass spectrometry method that is increasingly paired with NMR to associate the degree of modification within a specific population (fraction) or even individual macromolecules [145]. However, it also has limitations associated with the choice of matrix and the inability of the high molecular mass fractions to fly. Thus, despite recent advances, more research is needed to improve the analytical precision and characterization efficiency of those natural polymers and macromolecules incorporated in the hybrid networks.

## 9. Conclusions

Significant research efforts to reduce humans’ ecological footprint have produced an increasing number of hybrid networks containing one or more natural polymers and macromolecules. Despite the dispersity in size and molecular mass of the natural components (a fundamental limitation), they display new or improved properties that render them attractive for a broad array of applications, including but not limited to environmental cleanup, biotechnology, the food industry, and medicine. Two new directions are currently emerging in this area of study. The first one is the search for new bioderived building blocks. While most studies utilize the polymers discussed in this review—cellulose, chitin, alginic acid, gellan gum, and lignin—more efforts are being devoted to the exploration of other natural macromolecules such as starch [146], hyaluronic acid [147], and spider silk [148,149]. Many of those studies employ environmentally friendly solvents, low energy, and biocatalysis, which constitute the second direction for the utilization of “green” cross-linking processes. The work by Kaplan’s group and others is a good illustrative example of the attempts in this area [150,151]. Additive manufacturing is the latest innovative technology that will greatly improve the synthesis of multifunctional polymer networks. Due to increasing speed and printing resolution, a new generation of bioinks is expected to emerge shortly [152]. Three-dimensional printing will be broadly used to generate hybrid networks in three-dimensional form as analogs or semi-artificial substitutes for human organs and tissues [153].

All these recent developments trace a tendency of ever-increasing usage of natural polymers and macromolecules and their incorporation in new versatile networks through innovative, environmentally friendly technologies.

## Figures and Tables

**Table 1:** Additional examples of sustainable networks, their synthesis, and applications.

Natural Precursor	Potential Use	Synthesis Method	Reference
Cellulose and locust bean gum	3D printing	Suspensions of locust bean gum and cellulose microfibers are mixed and homogenized. Prepared hydrogel is inserted into Luer Lock syringes and refrigerated for complete gelation. Inks are printed using a 3D bioprinter and stored at ambient temperature.	[124]
Chitin	Potential tissue engineering	Chitin is dissolved in an 8% NaOH/6% urea solution using the freezing/thawing method and then blended with sodium alginate solution, attapulgite powder, and epichlorohydrin.	[125]
Chitin and alginate	Wound healing	Self-healing hydrogels are prepared by cross-linking acrylamide-modified β-chitin (Am-β-Chn) with alginate dialdehyde (AD). Chitin solution (2%) is prepared, and a fixed volume of various AD concentrations are added to yield pre-hydrogel mixtures. Solutions are left to gel completely.	[126]
Gellan gum (GG)	Tumor therapy and bone reconstruction	GG is dissolved in deionized water at 80 °C. Graphene oxide is dispersed in deionized water using ultrasonication. Bio-inks are formed by mixing GG and GO aqueous solutions in different concentrations. Prepared GG/GO 3D scaffolds are then soaked in CaCl_2_ solution. Final hydrogels are obtained after freeze-drying.	[127]
Gellan gum	Sustained drug release	Spherical diclofenac sodium-loaded carboxymethyl tamarind gum-GG IPN microbeads are prepared using AlCl_3_ as an ionic cross-linker. Aqueous GG and CTG solutions are mixed, and the diclofenac sodium is added. The homogeneous drug–polymer mixtures are added dropwise into 3% w/v AlCl_3_ solution, resulting in the formation of rigid spherical microbeads.	[128]
Gellan gum and cellulose	3D bioprinting of skin cells	Hydrogel formulations are prepared by mixing GG water solution and a suspension of nano-fibrillated cellulose. Hydrogels are fully cross-linked in 30 min by immersion in a CaCl_2_ 1% (w/v) bath for mechanical characterization.	[129]
Polylactic acid, chitosan, cellulose nanocrystals, and ethyl lauryl alginate	Antibacterial food packaging material	An antibacterial food packaging material with a trilayer structure is obtained through the combination of extrusion, coaxial electrospinning, and coating techniques.	[130]
Cellulose nanofiber/polyvinyl alcohol with aldehyde cellulose nanofiber as cross-linker	Highly temperature-resistant hydrogels	Cellulose nanofibers and polyvinyl alcohol are dispersed into water and stirred. HNO_3_ catalyst is added to the mixture, and the hydrogel precursor is transferred into a sealed transparent mold. Hydrogel is placed in an oven for 24 h.	[131]
Sodium alginate and carboxymethyl cellulose	Material with excellent water-induced shape memory properties	A dual-crosslinked gel is formed by sodium alginate reaction with glutaraldehyde (GA) and Ca^2+^. Carboxymethyl cellulose (CMC) is introduced into the cross-linked network.	[132]
Lignin and polyurethane	Tough, thin, printable hydrogels	Lignin is used as a toughening component for polyether-based polyurethane (HPU) hydrogels. Various concentrations (0, 0.5, 1, and 2.5 wt%) are prepared from lignin stock solutions and added to HPU at pH 7–8. Samples are cast and dried at 60 °C for 3 days.	[133]
Lignin, poly(ethylene glycol) methyl ether methacrylate (PEGMA)-grafted lignin	Mechanically responsive and self-healing hydrogels	Hyperbranched copolymers are prepared by atom transfer radical polymerization to form supramolecular hydrogels with a very low critical gelation concentration of 1 wt% in the presence of α-cyclodextrin.	[134]

## Data Availability

No new data were created or analyzed in this study. Data sharing is not applicable to this article.

## Sample Availability

No samples were created or analyzed in this study. Sample availability is not applicable to this article.
